# Single-cell tumor microenvironment profiling informs a circulating proteome test for the interception of malignant transformation in NF1 nerve sheath tumors

**DOI:** 10.21203/rs.3.rs-6865989/v1

**Published:** 2025-06-18

**Authors:** Jack Shern

**Affiliations:** National Cancer Institute

**Keywords:** Neurofibromatosis type 1, NF1, nerve sheath tumor, malignant transformation, single-cell sequencing, single-cell tumor atlas, plasma proteomics, biomarker

## Abstract

The most common cause of death in neurofibromatosis type 1 (NF1) is the development of malignant peripheral nerve sheath tumor (MPNST), a deadly sarcoma that can transform from benign plexiform neurofibromas (PN) or premalignant atypical neurofibromas (AN). We built a single-cell dataset of 55 NF1-associated PN, AN, and MPNST to define cellular changes in neurofibroma at-risk of malignant transformation. Integrative analysis of changes in the tumor microenvironment revealed the emergence of malignant tumor cells, regulatory T cells, and loss of activated macrophages in MPNST. Using this reference dataset, we validated findings using anchor-based label transfer in an additional 19 NF1 nerve sheath tumors profiled with single cell sequencing, and public datasets. We then defined protein biomarkers of malignant transformation from high-throughput proteomic analysis of plasma samples collected from 45 NF1 patients that correlated to mRNAs specific to MPNST cell populations. Fifty plasma proteins accurately and non-invasively distinguished patients with MPNST from those with premalignant tumors. These markers should improve the ability to identify high-risk neurofibromas for improved cancer surveillance and enable early detection of malignant transformation in NF1.

## ONE SENTENCE SUMMARY

Single-cell tumor sequencing of the tumor microenvironment and high-throughput plasma proteomics of NF1 patients were used to accurately differentiate malignant peripheral nerve sheath tumors from their benign precursors.

## INTRODUCTION

Neurofibromatosis type 1 (NF1) is the most common autosomal dominant cancer predisposition syndrome and is caused by a germline mutation of the tumor suppressor gene *NF1*^1^. Approximately 1/3000 individuals are born with NF1, and the population is nearly 10 times more likely to develop cancer than the general population^2^. In particular, NF1 is associated with the development of malignant peripheral nerve sheath tumors (MPNST) at an early age and with a 1000-fold higher risk compared to the general population^2,3^. In the setting of NF1, MPNST typically arise within benign plexiform neurofibroma (PN) and frequently transition through a premalignant condition called a atypical neurofibroma (AN) or Atypical Neurofibromatous Neoplasm with Uncertain Biologic Potential (ANNUBP)^4^. MPNST are refractory to both chemotherapy and radiation therapy^5,6^. For potential cure of MPNST, complete surgical resection with wide negative margins is required, however, this requires early diagnosis of localized disease or identification of premalignant lesions, which can be resected with narrow margins^7,8^. It is therefore critical to understand the mechanisms of malignant transformation and define biomarkers that identify at-risk tumors before they become cancerous.

The sensitivity of clinical, histopathologic and radiographic diagnostics for MPNST is hindered by significant intratumoral heterogeneity that challenges detection of localized, early disease that would be most amenable to curative intent resection. Single-cell sequencing has emerged as a powerful tool for dissecting the complex cellular heterogeneity in both healthy tissues and neoplasms. Single-cell RNA sequencing (scRNAseq) and single nuclei RNA sequencing (snRNAseq) have been used to profile the intratumoral heterogeneity of human and mouse PN^9,10^, human AN^11^, and human MPNST^12,13^. These studies identified a complex cellular tumor microenvironment (TME) that is likely to have functional consequences on tumor development and growth.

To advance the understanding of the TME changes that accompany malignant transformation and deliver clinically relevant biomarkers of this process, we built a precancer tumor reference dataset that integrates scRNAseq of clinically annotated patient specimens collected from peripheral nerve sheath tumors of each pathologic stage. The dataset was used to define unique immune, stromal, and tumor cellular populations that appear in the TME over the course of malignant transformation and validated a method that identifies transforming cells in an independent cohort of clinically concerning cases. We leveraged the novel TME dataset by coupling it with high-throughput plasma proteomics to define a group of 50 proteins that accurately classified patients with MPNST. Our approach demonstrates improved accuracy for detection of high-risk NF1 nerve sheath tumors that may undergo malignant transformation and it can be translated to the clinic for early diagnosis and possibly prevention of MPNST.

## RESULTS

### Transcriptomic profiling of the cellular composition of NF1 nerve sheath tumors

**Figure 1:**
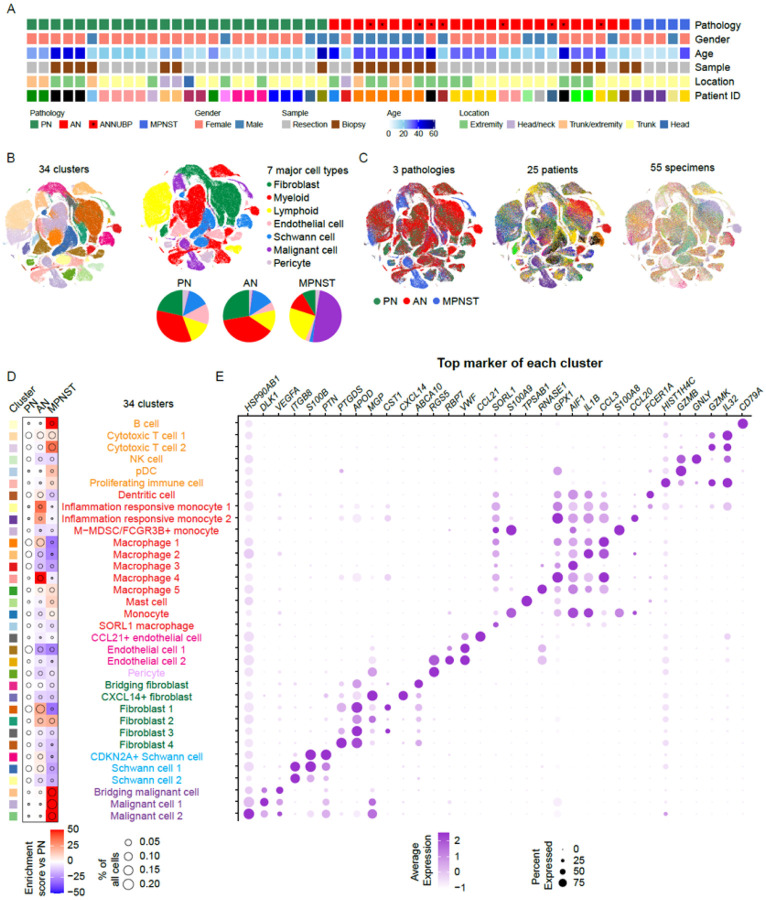
Cohort and definition of cell types and composition in the NF1 nerve sheath tumor microenvironment. **A.** Pathological diagnosis and clinical characteristics of the profiled nerve sheath tumors included in the scRNAseq dataset. **B.** t-distributed stochastic neighbor embedding (tSNE) plots of all cells by major cell types (left panel) and 34 major cell clusters (right panel). **C.** tSNE plots of all cells color-coded by tumor types (first panel), individual patient (second panel), and specimens (third panel). **D.** Compositional alterations in all 34 major cell clusters in ANF and MPNST in comparison to PNF. **E.** Dot plot showing the expression of the top marker gene of each cell clusters.

To build a reference single-cell dataset of NF1-associated peripheral nerve sheath tumors, we performed scRNAseq in clinically annotated NF1 tumors that were collected between January 2018 and April 2022. The cohort included 55 clinical specimens collected in 30 surgeries from 25 patients (Fig. 1A, **Supplementary Table 1**). The samples were resected tumors from debulking surgeries (n = 36) or tissue cores from needle biopsies (n = 19). Histopathologic diagnoses were determined by the National Cancer Institute Laboratory of Pathology. Among the 55 specimens, 25 were plexiform neurofibroma (PN), 25 were atypical neurofibroma (AN), and 5 were malignant peripheral nerve sheath tumors (MPNST). AN was defined by the presence of one of the following criteria: cytologic atypia, loss of neurofibroma architecture, hypercellularity, or a mitotic index > 1/50 but < 3/10 high-power fields. If an AN exhibited two of these features, it was diagnosed as atypical neurofibromatous neoplasm of uncertain biologic potential (ANNUBP)^4^. In the cohort, 9/25 AN met the criteria of a ANNUBP (Fig. 1A, **Supplementary Table 1**). Given the limited numbers of ANNUBP in the cohort, for this study, we grouped AN and ANNUBP together, restricting our comparisons to PN, AN, and MPNST.

After quality control and removal of low-quality cells (see Materials and Methods), integrative analysis was performed on a total of 421,377 cells. The study included 132,446 cells from 25 PN, 214,522 cells from 25 AN, and 74,409 cells from 5 high-grade MPNST (Fig. 1B). After dimension reduction, the cells were divided into 34 distinct transcriptional clusters (Fig. 1B, **left panel**) that could be assigned to one of seven major cellular compartments: fibroblast, myeloid immune cells, lymphoid immune cells, endothelial cells, Schwann cells, malignant cells, and pericytes (Fig. 1B, **right panel**). Cell proportions were evenly distributed among the three pathological stages except for the MPNST-specific malignant cells (Fig. 1C, **first panel**). Additionally, cell distributions among different patients (Fig. 1C, **second panel**) and different specimens (Fig. 1C, **third panel**) were consistent. Across the dataset, myeloid immune cells including macrophages and dendritic cells were the most abundant cell type, accounting for 31.9% of all cells (34.1% PN, 37.9% AN, 11.5% MPNST). Fibroblasts were the second most abundant cell type comprising 22.3% of the entire dataset (21.5% PN, 27.6% AN, 8.6% MPNST). Schwann cells comprised 11.4% of all cells (13.0% PN, 13.6% AN, 2.1% MPNST) and endothelial cells 7.1% (13% PN, 5.1% AN, 2.6% MPNST). Comparisons across pathologic states revealed relative contraction of myeloid immune cells and fibroblasts in MPNST when compared to PN, with no notable differences between PN and AN. The percentage of total endothelial cells decreased as the tumors underwent malignant transformation from benign PN through AN to MPNST. Two cellular compartments increased in MPNST compared to PN: lymphoid immune cells (15.5% of total, 14.2% PN, 13.3% AN, 23.6% MPNST) and malignant cells (9.0% of total, 0.4% PN, 0.5% AN, 49.0% MPNST). Notably, in several samples histologically diagnosed as PN and AN, we detected small populations of cells clustering with malignant cells, potentially marking these as tumors at risk of transformation.

Marker genes with the highest expression were obtained from each cell cluster and the cell types were annotated by comparing the top marker genes to canonical cell markers^9,12^. Comparisons between pathologic diagnosis by Pearson Residual (see Materials and Methods) enabled calculation of an enrichment score for each cluster (Fig. 1D) and the expression of the top unique marker gene of each cell cluster was plotted (Fig. 1E). MPNST had a reduction of multiple macrophages subtypes, fibroblasts, and Schwann cells (Fig. 1D). Lymphoid immune cells including B cells, cytotoxic T cells, and plasmacytoid dendritic cells (pDCs) increased as a percentage of all cells in MPNST when compared to PN. Interestingly, while two clusters of macrophages slightly decreased in percentage in AN, inflammation responsive monocytes, macrophage 1, and macrophage 4 (marker gene *GPX1*, Fig. 1E) showed proportional increases in AN when compared to PN. Notably, unique immune cell populations, such as regulatory T cells (Tregs) that were reported to be part of the TME of MPNST^12^, were absent from this high-level analysis. This observation indicated further subclustering was required to define additional cellular changes in the TME associated with malignant transformation.

### Immune TME changes associated with malignant transformation

**Figure 2:**
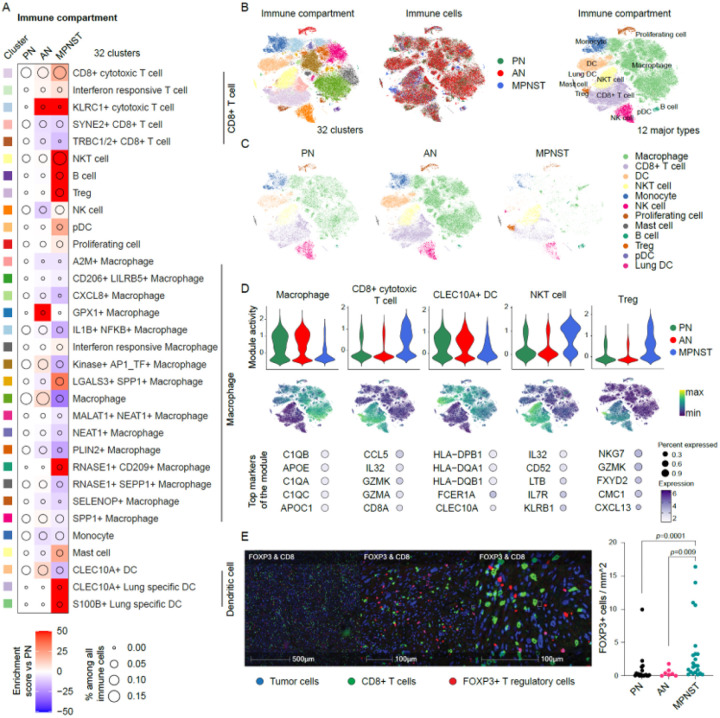
The immune compartment in NF1 nerve sheath tumors. **A.** Compositional alterations in 32 immune cell clusters in ANF and MPNST relative to PNF. **B.** tSNE plots of immune cells by 32 clusters (left), tumor type (middle), and 12 major immune cell types (right). **C.** tSNE plots of immune cells by 12 major immune cell types presented separately by tumor types. **D.** Immune gene program activity shown in violin plots and tSNE plots. The expression levels of the top 5 markers of these clusters were shown in dotplots. **E.** Representative pictures (left panel) of MPNST specimens stained with immunohistochemistry and deconvoluted with HALO analysis software to overlay serial sections that display nuclei (blue), CD8+ staining (green), and FOXP3+ staining (red). Results of the quantification and statistical analysis shown in the dot plot (right panel) indicates the enrichment of FOXP3+ regulatory T cells in MPNST samples.

To further dissect the cellular composition of the tumor immune compartment, we subclustered the 196,671 cells that were annotated as immune cells (including myeloid and lymphoid immune cells). These cells comprised 62,113 cells (31.6%) from PN, 108,619 cells (55.2%) from AN, and 25,939 cells (12.2%) from MPNST. The analysis enabled the identification of 32 transcriptionally distinct clusters, including natural killer (NK) cells, NKT cells, Tregs, B cells, pDC, monocytes, mast cells, 5 different CD8 + T cell populations, 16 different macrophage populations, and 3 different DC clusters (Fig. 2A). Broadly, these clusters were attributed to 12 major immune cell types (Fig. 2B).

Immune cellular composition was largely unchanged between PN and AN. Notably, however, AN had relative increases in the proportions of cytotoxic T cells expressing high levels of *KLRC1* and macrophages expressing high levels of *GPX1* (Fig. 2A). *KLRC1* (or *NKG2A*) is an inhibitory receptor for both CD8 + cytotoxic T cells and NK cells and is predominantly expressed by CD8 + T cells in human lung cancer where their presence is correlated with the progression of chronic infection and cancer^14^.

In contrast, comparisons of the immune cellular composition of PN and MPNST revealed multiple differences. First, the macrophage compartment contracted in MPNST when compared with PN, with two exceptions: MPNST demonstrated an increased proportion of LGALS3 + SPP1 + macrophages and RNASE1 + CD209 + macrophages (Fig. 2A). These macrophage subtypes are known to be associated with immune suppression in the TME^15^. Second, lung-specific DC were unique to MPNST (Fig. 2C). Given that 40% of the MPNST samples were metastatic tumors collected from the lung, the identification of CLEC10A + and S100B + lung specific DC was expected. Finally, Tregs, marked by expressions of *LAG3* and *CTLA4*, and consistent with those described in an early dysfunctional state^16^, were uniquely present in MPNST tumors (Fig. 2D). The presence of FOXP3 positive Tregs was confirmed by immunohistochemistry staining and analysis in an independent cohort of MPNST tumors (Fig. 2E).

### Increase of TAGLN + and RGS5 + pericytes in the MPNST TME

**Figure 3:**
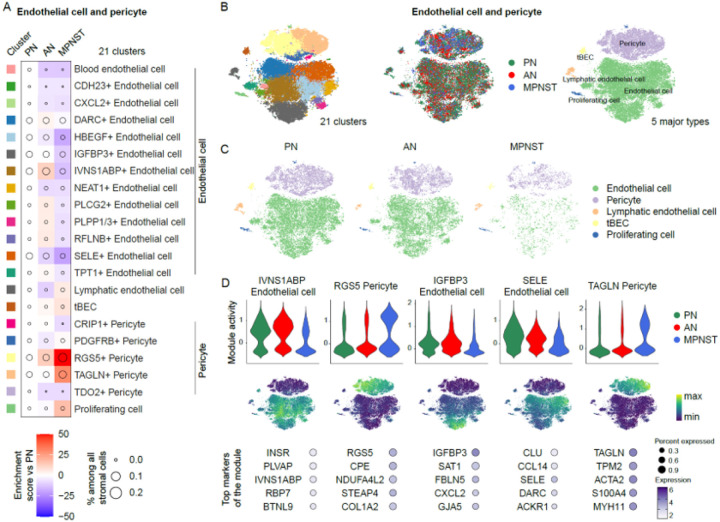
Vasculature in the TME of NF1 nerve sheath tumors. **A.** Compositional changes in 22 endothelial cell and pericyte clusters in ANF and MPNST relative to PNF. **B.** tSNE plots of endothelial cells and pericytes by 22 cell clusters (left), tumor type (middle), and 5 major cell types (right). **C.** tSNE plots of endothelial cells and pericytes by 5 major cell types presented separately by tumor types. **D.** Marker gene program activity shown in violin plots and tSNE plots. The expression levels of the top 5 markers of these clusters were shown in dotplots.

Next, cells annotated as pericytes and endothelial cells were independently subclustered. Clustering of 40,269 cells (21,704 PN, 14,760 AN, and 3,805 MPNST) yielded 21 distinct clusters; including a lymphatic endothelial cell population, a tip blood endothelial cell (tBEC) population, a proliferating cell population, 13 unique endothelial populations, and 5 pericyte populations (Fig. 3A). These clusters were grouped into 5 major cell types: endothelial cells, lymphatic endothelial cells, tBEC, pericytes, and proliferating cells (Fig. 3B). While all blood endothelial cell clusters in MPNST decreased in proportions when compared with PN, lymphatic endothelial cells and tBEC proportionally increased in the MPNST samples (Fig. 3A). Interestingly, the TME of AN and MPNST were enriched for pericytes relative to PN. RGS5 + pericytes, specifically, increased in proportion as the tumors underwent malignant transformation from benign PN through AN to MPNST (Fig. 3C).

Module activities, defined as the average expression for the top 50 marker genes, were significantly elevated for RGS5 + pericytes and TAGLN + pericytes in MPNST cells as compared to cells from PN and AN (Fig. 3D). Additionally, the alterations among the three pathologies in module activities of other endothelial cell types (shown in representative figures for IVNS1ABP + endothelial cells, IGFBP3 + endothelial cells, and SELE + endothelial cells) were consistent with the observed cell proportional changes among the pathological stages (Fig. 3D).

### Aneuploidy marks malignant cells and a population of “bridging” cells

To accurately identify MPNST cells, inferCNV was used to mark specific patterns of aneuploidy in three cellular clusters annotated as malignant cells in the single-cell reference dataset (**Supplementary Fig. 1A**)^17^. To ensure consistency, inferCNV was also used on all cells of the Schwann, fibroblast, and malignant cell clusters, which led to the accurate grouping of cells separated by their distinct patterns of inferred CNV (**Supplementary Fig. 1B**). Cells from clusters “Malignant cell 1” (**Supplementary Fig. 2A**) and “Malignant cell 2” (**Supplementary Fig. 2B**) exhibited distinct CNV patterns consistent with the bulk sequencing of the matched MPNST tumor samples. In contrast, while the majority of the cells in the “Bridging malignant cell” cluster exhibited MPNST-specific inferred CNV patterns, a small proportion of cells did not demonstrate any MPNST-specific CNV (**Supplementary Fig. 2C**). To define these cells with uncertain CNV patterns, we performed hierarchical clustering of cells from the “Bridging malignant cell” cluster and this analysis yielded 8 groups (color bar on the left of Supplementary Fig. 2C). Analysis of the samples contributing cells to these groups revealed that only cells from histologically confirmed MPNST samples comprised cellular groups 1–7 (10,370 out of 11,611 cells, 89.3%, **Supplementary Fig. 2D**). Interestingly, cells that defined group 8 showed diverse CNV patterns and were derived not only from patients with MPNST but also patients with PN and AN (1,241 out of 11,611 cells, 10.7%, **Supplementary Fig. 2D**), suggesting that group 8 may represent cells at risk for transforming from benign or pre-cancerous to malignant. Marker genes that were highly expressed in this group of cells include the cancer-associated imprinted genes *XIST, NEAT1*, and *MEG3*.

### Prediction of malignant transformation from scRNAseq-guided plasma protein biomarkers

Fine needle tissue biopsies have a high positive predictive value for MPNST, however, the sensitivity of tissue biopsy in a highly heterogenous tumor is limited by geographic survey bias^18^. Liquid biopsy using cell free DNA (cfDNA) has previously been shown to circumvent challenges of geographic heterogeneity in NF1 to accurately and non-invasively distinguish between PN, AN, and MPNST^19,20^. Previous studies, however, have demonstrated that many early-stage cancers are associated with circulating tumor DNA (ctDNA) fractions below the limits of detection of standard next generation sequencing analyses and that circulating proteins may be more abundant biomarkers of early disease^21–23^. We therefore hypothesized that our scRNAseq TME insights into the NF1 nerve tumor transcriptome could inform a highly specific, non-invasive circulating proteomic liquid biopsy assay to mitigate the challenges of tissue heterogeneity and enable early MPNST interception. To accomplish this, we first performed a broad survey of the circulating proteome, applying a high-throughput protein extension assay (PEA)^24^ to 118 plasma samples from healthy volunteers (*n* = 10) and patients with NF1 and a spectrum of peripheral nerve sheath tumors (PN (*n* = 29), AN (*n* = 25), and MPNST (*n* = 54), **Supplementary Table 3**). The dual-recognition antibody assay^25^ resulted in normalized protein expression (NPX) for 1,436 proteins, 595 of which were found to be differentially expressed (log2FC > 0, *p* adj < 0.05) in the plasma of patients with MPNST relative to the plasma of patients with PN by ANOVA and post-hoc Tukey Honestly Significant Difference (HSD) (**Supplementary Fig. 4A**). Leveraging the cell-type specific marker genes from our NF1 single-cell tumor reference dataset (MPNST vs PN enrichment score > 25), we identified 127 MPNST gene markers whose encoded protein was also assayed on the PEA antibody panel (Fig. 6A; **Supplementary Fig. 4A**). These 127 genes were highly expressed in multiple single-cell clusters (**Supplementary Fig. 4B**). Gene ontology (GO) functional analysis (*38*) demonstrated significant enrichment of genes involved in inflammatory response (false discovery rate (FDR) = 5.4e-5), chemotaxis (FDR = 5.7e-5), positive regulation of ERK1 and ERK2 (FDR = 3.6e-4) and positive regulation of angiogenesis (FDR = 9.8e-3) (Fig. 6B; **Supplementary Table 4**). Forward selection of statistically elevated circulating proteins (*n* = 595) by PEA-represented MPNST marker genes (*n* = 127) resulted in a final list of 50 scRNAseq-informed circulating protein biomarkers representing MPNST TME specific clusters (Fig. 6C). Projection of these 50 selected genes onto scRNAseq tSNE plot highlighted MPNST tumor cells, MPNST-specific fibroblasts, and MPNST-specific immune subclusters (Fig. 6D).

**Figure 4:**
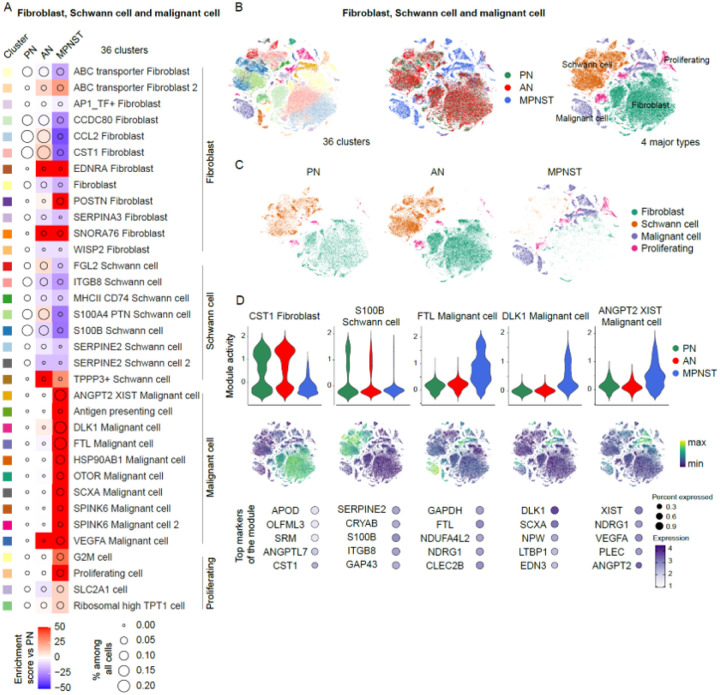
TME profiling of fibroblast and tumor compartments of NF1 nerve tumors. **A.** Compositional changes in 36 fibroblast, Schwann cell, and malignant cell clusters in ANF and MPNST relative to PNF. **B.** tSNE plots of fibroblasts, Schwann cells, and malignant cells by 36 cell clusters (left), tumor types (middle), and 4 major cell types (right). **C.** tSNE plots of fibroblasts, Schwann cells, and malignant cells by 4 major cell types presented separately by tumor types. **D.** Marker gene program activity shown in violin plots and tSNE plots. The expression levels of the top 5 markers of these clusters were shown in dotplots.

**Figure 6:**
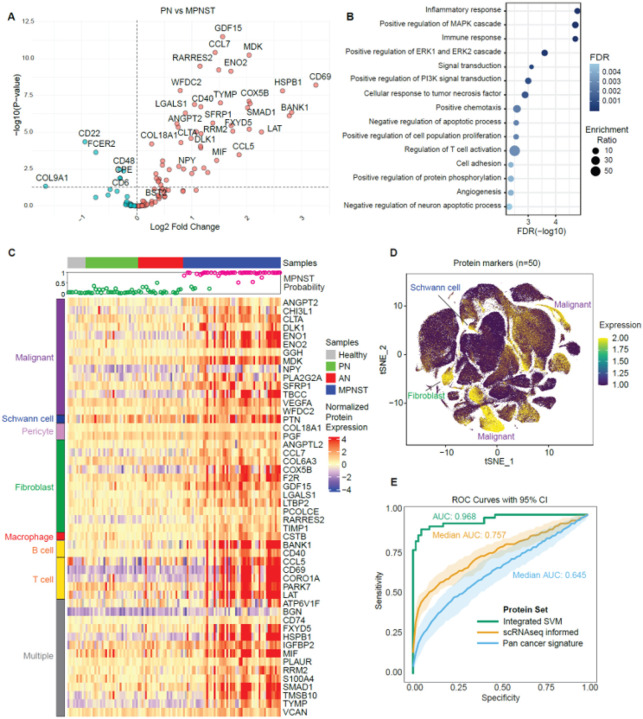
scRNAseq-informed plasma protein markers distinguished patients with MPNST from patients with benign peripheral nerve sheath tumors. **A.** Volcano plot of relative plasma protein expression for 127 scRNAseq-identified candidate MPNST markers. x-axis is log2 fold change of protein expression, y-axis is −log10(P-value) comparing plasma from patients with MPNST versus plasma from patients with only PN (see Materials and Methods). **B.** Gene ontology (GO) functional analysis of 127 scRNAseq-identified candidate MPNST protein markers. The 15 most significantly enriched GO terms are presented with the false discovery rate (FDR) and enrichment ratios. **C.** Heatmap of normalized protein expression for 50 proteins over-expressed in MPNST plasma and highly enriched (enrichment score > 25) in MPNST TME scRNAseq clusters. One hundred of eighteen blood samples are organized in columns by clinical diagnosis, 50 candidate MPNST protein biomarkers are listed in rows by their predominant scRNAseq cell cluster. Inset MPNST Probability visualizes the 50-protein integrated SVM model’s median predicted probability that a sample is MPNST (≥0.5 probability, pink) or other (<0.5 probability, green) in one-versus-all comparison. **D.** tSNE plot overlaying the average expression of 50 nominated MPNST protein markers on scRNAseq cell clusters (see Materials and Methods). **E.** Receiver operating characteristic (ROC) curves of 50-protein SVM integrated model’s (green line), 50 individual scRNAseq informed plasma proteins’ (median ROC curve (orange line), and 95% confidence interval (CI) (orange ribbon)), and individual proteins from a previously published pan-cancer plasma protein panel’s (median ROC curve (blue line), 95% CI (blue ribbon)) performance differentiating MPNST from others in one-versus-all comparisons.

Individually, each of the 50 scRNAseq selected proteins distinguished MPNST samples in one-versus-all (OVA) comparisons with high specificity (median specificity 0.90 (IQR 0.82–0.94), median sensitivity 0.60 (IQR 0.52–0.71), median AUC 0.76 (IQR 0.71–0.8), Fig. 6E; **Supplementary Fig. 5**). Importantly, our method identified DLK1, a marker gene highly expressed in malignant cells (Fig. 4D), as a plasma protein marker (Fig. 6C, **Supplementary Fig. 5**) for MPNST. This observation is consistent with the recent report of DLK1 as a biomarker whose overexpression marked immunosuppressive TME and worse overall survival in patients with MPNST^10^. We next assessed whether scRNAseq *a priori* knowledge improved predictive power of MPNST-specific protein markers relative to published pan-cancer protein biomarkers. Our dataset was filtered for individual circulating proteins described in a recently published analysis of plasma proteins associated with 19 different cancers^26^. These published pan-cancer proteins, individually, were inferior in detecting MPNST (median specificity 0.80 (IQR 0.63–0.89), median sensitivity 0.57 (IQR 0.43–0.70), median AUC 0.65 (IQR 0.57–0.72) relative to our MPNST-specific scRNAseq informed candidate proteins.

We next integrated all 50 protein markers into a single model using a 10 repeat, 5-fold cross-validation (CV) support vector machines (SVMs) method (see Materials and Methods). Using the integrated protein model, the median probability of 10-repeats for each sample being an MPNST (OVA comparison) was termed the MPNST probability score. Performance of the integrated protein model was assessed using Youden’s Index and a receiver operating characteristic (ROC) curve of MPNST probability OVA scores. Indeed, integration of all 50 proteins improved our assay’s performance (specificity 1.0, sensitivity 0.76, AUC 0.97) and accurately distinguished MPNST from healthy volunteers, PN and AN (Fig. 6C).

## DISCUSSION

To our knowledge this study represents the largest collection of single-cell transcriptomic and plasma proteomic data of NF1 patients to date, offering an unprecedented examination of the cellular composition changes that underlie malignant transformation in this condition. In addition to describing the TME changes that occur as these tumors evolve, we leveraged this dataset to develop high-throughput single-cell and plasma proteomic methods that enable early and accurate clinical detection of MPNST. Our novel approach moderates the significant challenge of heterogeneity in these tumors and offers a leap forward in personalized disease management. We anticipate that these findings will further enable opportunities to develop additional highly accurate multimodal tests that incorporate advances in radiographic early detection^27^, cell free DNA detection^19,20^, and digital pathology for patients with NF1. Our methodology, built upon this unique NF1 dataset, may also extend to other cancer predisposition syndromes where early cancer detection of malignant transformation is critical.

This study significantly advances the current understanding of the TME compositional changes along the malignant transformation trajectory in NF1, including notable changes of the immune, stromal, and tumorigenic cells. Immune therapies represent potential novel treatment strategies for MPNST and the poor understanding of the mechanisms that enforce the immunosuppressive TME of MPNST tumors is an area of active investigation. There are case reports of MPNST patient responses to immune checkpoint therapies^28,29^ and MPNSTs have also been found to upregulate PD-L1 and lack PD1 expression, suggesting a mechanism for immune evasion that could be targeted by checkpoint inhibitors^30^. The therapeutic relevance of these observations is formally being tested in an ongoing clinical trial (NCT04465643) of nivolumab (PD-L1 inhibitor) plus ipilimumab (targeting CTLA4). Our data advances this field of investigation by highlighting the emergence of multiple cell types characteristic of an immunosuppressive MPNST TME including LAG3 + CTLA4 + Tregs, CD27 + B cells, and SPP + macrophages. Previous work has shown that MPNST tumor grade is correlated with the presence or absence of cytotoxic T lymphocytes (CD8+, CD4+, FOXP3+, CD45RO+, and CD56)^31^. Interestingly, we also observed elevation of tumor-associated T cell markers within the plasma of MPNST patients, nominating these cells as potential biomarkers of malignant transformation.

Our work uncovered additional MPNST specific cellular populations including a rare population of MPNST specific B cells characterized by the expression of CD27, TGFB1, and loss of IL1b. While these cells lacked expression of cytokines typical of B regulatory cells (IL10, IL27), there is growing evidence of the complex role of tumor associated B cells in response to immune checkpoint blockade^32^. We also discovered the emergence in MPNST of SPP1 + macrophages which have been implicated in fibrogenesis^33^, prognosis^34^ and metastasis^35^ in other tumor types. Our finding that 35% of the cells in benign tumors were macrophages is consistent with multiple reports that demonstrated up to 40–60% of cells from the benign PN and precancerous AN are macrophages^9,11,36^. In addition, recent work has demonstrated a predominance of CD163 + myeloid cells within the MPNST TME^37^. Future efforts should be dedicated to understanding the potential regulatory role of these cells in nerve sheath tumors, and how myeloid-modulating therapeutic strategies might be employed to target the TME.

Beyond the immune composition changes, we identified alterations in the stromal components of the NF1 TME over the course of malignant transformation. These included the reduction of endothelial cells in MPNST and the induction of pericytes from PN to MPNST. A notable finding was the increase in RGS5 + pericytes in MPNST. Regulator-of-G-protein-signaling-5 (RGS5) is a specific marker of pericytes, which play a critical role in the homeostasis of blood vessel formation and maintenance^38^. In mice, high expression of RGS5 is a marker of proangiogenic cells and its expression serves as a marker of tumor-associated pericytes^39^. Expression of RGS5 in tumors is correlated with tumor growth, metastasis and poor prognosis^40,41^ and high RGS5 levels in tumor pericytes leads to an immunosuppressive TME^42^. In addition, these pericytes are crucial to tumor neo-angiogenesis and elevated expression of RGS5 contributes to cellular survival within the TME^43^. Accordingly, loss of RGS5 promotes pericyte-maturation, morphologically normalizes the vasculature, and improves immune infiltration within the TME^44^. It is possible that the presence of these cells in the malignant TME is correlated with the evidence of increased angiogenesis that we identified in the plasma proteomics of MPNST patients, where ANGPT2, CD40, CHI3L1, HSPB1, PGF, and VEGFA were found to be biomarkers of malignancy. The presence of these cells may also have important therapeutic implications. A recent study profiling the vascular TME across more than 10,000 solid tumors of the TCGA revealed that high levels of RGS5 was a marker of a subgroup of tumors predicted to respond to bevacizumab, due to a vascular TME signature indicating a denser vessel network^45^. While the clinical benefit rate of a trial of 25 patients with MPNST using the combination of everolimus (mTOR inhibitor) and bevacizumab (VEGF inhibitor) was only 12%^46^, deeper understanding of the role of RGS5 + cells in metastasis and immunomodulation of the MPNST TME may provide novel therapeutic avenues.

The most profound cellular composition changes in the TME were associated with the tumorigenic Schwann cells that are the cell-of-origin of NF1 nerve tumors^47,48^. Genetically, the transition driving the PN to AN transformation is loss of *CDKN2A* in the *NF1*-deficient Schwann cells^49–51^. Interestingly, in this study, we identified a large Schwann cell population in benign PN that express high levels of *CDKN2A*. Neoplastic Schwann cells under oncogenic pressure of hyper-active RAS are known to upregulate CDKN2A as a senescence mechanism to avoid malignant transformation^51^. We defined a transcriptional program characteristic of MPNST cells including highly expressed marker genes characteristic of these malignant cells include *DLK1, GAPDH, FTL*, and *NDRG1*, among others. We also found a unique “bridging” cell population in some benign tumors whose transcriptome had some similarity to the profile of MPNST cells, perhaps identifying them as at risk of malignant transformation. Unique marker genes of these cells included several long non-coding RNAs (lncRNA), such as *MEG3* (maternally expressed 3) and *XIST* (X-inactive specific transcript), which are both maternally expressed imprinted genes. XIST is carried on the X chromosome and responsible to initiate the X chromosome inactivation. One limitation of our study is the over-representation of females in our MPNST population. Nevertheless it is interesting that gene silencing on the inactive X chromosome is maintained by repressive epigenetic mechanisms including DNA methylation, polycomb repressive complex 2 (PRC2)-mediated histone modification, and direct interactions between XIST and RNA binding proteins^52^. Human *XIST* is known to interact with epigenetic modifiers such as PRC2^53^ and the interaction between PRC2 and the XIST RNA molecule is critical for regulation of X chromosome inactivation. Importantly, through sequencing analysis in patient samples, loss of PRC2 and its function in properly maintaining global transcriptional repression has been reported in ~ 80% of all MPNSTs^54,55^. Therefore, it is possible that abnormal elevation of XIST expression that we observed is correlated with the loss of functional PRC2.

Precancer atlases for multiple epithelial cancers have been reported including sporadic and familial adenomatous polyposis (FAP) colorectal cancer^56,57^, melanoma^58^, and breast cancer^59,60^ providing insights into oncogenic mechanisms and targets that might intercept or prevent those cancers. This study is the first precancer atlas reported for a sarcoma and highlights the translational impact of high-resolution profiling of the TME. While this impact is especially important in the context of early detection in a cancer predisposition population, there are several limitations of the study. The number of samples, while large for a rare tumor type, is likely not inclusive enough to broadly capture patient to patient tumor variability. In addition, the spatial context and the functional consequences of many of the observed cellular populations within the TME will need to be validated in future studies. Despite these limitations, our work adds clarity to the understanding of the complex TME in NF1 nerve tumors. In addition, we demonstrate a novel application of scRNAseq-guided TME dissection by combining this information with circulating protein markers in plasma samples collected from NF1 patients. Excitingly, this approach nominated circulating protein markers that accurately anticipate malignant transformation and form the basis of a clinical tool that will benefit NF1 patients. Ultimately, this approach may facilitate the diagnosis and improve the clinical decision making required to improve outcomes in this hard-to-diagnose and hard-to-cure disease.

## METHODS

### NF1 patients for scRNAseq

Informed consent was obtained from all patients (or their legal guardians if the patients were minors). The collection of patient tumor samples, procedures for single-cell RNA sequencing, as well as data storage plans were approved by the National Cancer Institute Institutional Review Boards (NCT01109394; IRB identifier 10C0086). Our study examined males and females, and similar findings are reported for both sexes. All samples were collected with informed consent for research and IRB approval. Protocols are available on ClinicalTrials.gov.

### Healthy controls for plasma collection

After obtaining written consent, healthy donor blood samples were obtained at a single time point from appropriately consented donors at the NIH Department of Transfusion medicine (NIH protocol NCT00001846, NIH Intramural IRB identifier 99-CC-0168). Plasma samples were collected from a total of 10 healthy volunteers (**Supplementary Table 3**). Eligibility for healthy controls included age greater than 18 years old and no known history of neoplastic or hematological disorders. Protocols are available on ClinicalTrials.gov.

### NF1 patients for plasma collection and clinical classification

This study used blood samples prospectively collected from NF1 patients with PN, AN, and MPNST tumors. Patients from the NCI with clinically and radiographically diagnosed PN or pathology-proven AN and MPNST were enrolled with written informed consent (NCI protocol NCT01109394, NIH Intramural IRB identifier 10C0086; NCI protocol NCT00924196, NIH Intramural IRB identifier 08C0079) between 2016 and 2024. Additionally, MPNST samples from clinical trial SARC031 were included in analysis (NCT03433183). NF1 status was determined clinically by consensus criteria (78). A total of 29 PN, 25 AN, and 54 MPNST associated plasma samples were collected (**Supplementary Table 3**). AN and MPNST are defined by histological criteria^4^. Atypical neurofibromatosis neoplasm with unknown biological potential (ANNUBP) were grouped with AN for the included analyses. All patients underwent clinical management and follow-up per the standard-of-care. Protocols are available on ClinicalTrials.gov.

### Single-cell RNA sequencing

Procedures for the collection, banking, and process of patient NF1-associated peripheral nerve sheath tumors for single-cell RNA sequencing (scRNAseq) using a 10x Genomics platform were described previously^61^. Briefly, all patient tumor samples were directly collected from the operating room as a resected tumor from debulking surgery or as a core needle biopsy from ultrasound-guided diagnostic biopsy sampling surgery. In a laminar flow hood, resected tumor was minced into 1 mm^3^ cubes in Tumor Dissociation Media and transferred to a gentleMACS C Tube (Miltenyl Biotec, Bergisch Gladbach, Germany). Core needle biopsy did not need further mince before being placed in the Tumor Dissociation Media in a C Tube. Minced sample in the C Tube was first processed on a gentleMACS dissociator (Miltenyl Biotec) using the program “h_Tumor_2.1” twice followed by shaking at 200 rpm at 37°C for 40 min and then processed on the gentleMACS dissociator using the program “h_Tumor_2.1” once. The dissociated tumor sample was filtered through a 40 μm cell strainer and any remaining tumor chunks were pushed through the cell strainer and the cell strainer membrane was washed twice with DMEM. Filtered cells were washed with phosphate buffered saline (PBS) containing 0.04% bovine serum albumin (BSA) and collected by centrifugation at 300 x *g*. If the cell pellet appeared pink which was indicative of the existence of red blood cells (RBC), 10 mL of RBC Lysis Buffer (Sigma Aldrich) was used to resuspend and treat the cells at room temperature for 5 min. The cells were next washed twice with PBS containing 0.04% BSA and the cell viability and concentration were determined with the Propidium Iodide & Acridine Orange (AO/PI, Nexcelom, Lawrence, MA) staining in an automated fluorescent cell counter (Nexcelom). From each tumor sample, 2–4 capture lanes with a targeted 6000 live cells per lane to be captured were processed by on Chromium Controller (10x Genomics, Pleasanton, CA). We used either 3’ v2 or v3 reagents for library preparation following the manufacturer’s protocol. Specific version of reagent used and the number of capture lanes for each sample were documented in **Supplementary Table 1**. Sequencing of the cDNA library was performed on an Illumina NextSeq550 or NovaSeq 6000 sequencer, aiming to achieve at least 50000 reads per cell.

### Single-cell RNA sequencing data analysis of NF1 nerve tumors

#### Quality control and pre-processing

Raw sequencing data after base calling using RTA v3.9.2 was demultiplexed in Cell Ranger v6.0.0 using the embedded software Bcl2fastq v2.20.0. The demultiplexed fastq files were aligned to human genome GRCh37 in Cell Ranger v6.0.0 using the embedded STAR v2.7.2a. The generated count matrix from each capture lane was next corrected using SoupX^62^ to correct the ambient RNA contamination in the data with the contamination fraction set to 20%. The SoupX corrected count matrix from each capture lane was next processed in scrublet^63^ to estimate and label the predicted doublets based on an expected doublet rate and the calculated scrublet score for cells in each capture lane. Low quality cells (nFeature_RNA < 300 or > 7500, cells with mitochondrial gene content higher than 70%, as well as nCount_RNA < 500 or > 50000) and estimated doublets were removed before a count matrix was generated by merging the count matrices of all 114 capture lanes from 55 NF1 tumors in Seurat v4.0.4^63,64^ for downstream integration and analysis.

#### Data integration and annotation for single cell populations in NF1 tumors

After normalization, the top 5000 variable genes that are not mitochondrial genes or ribosomal genes were selected and used in downstream analysis. To correct for batch effect, the merged count matrix was integrated in Seurat v4.0.4 using the fastMNN wrapper^65^ with the setting of k = 20. The function RunUMAP in Seurat was next used to reduce dimensions with the setting of dims = 1:30. To identify distinct clusters, the Seurat function FindClusters was used with the settings of resolution = 0.8 and algorithm = 1, yielding a total of 35 cell clusters. Markers of each cluster were identified using the Seurat function FindAllMarkers in the “RNA” assay of the dataset with the setting of min.pct = 0.25. The marker genes of each cluster were assessed, and manual annotation was performed to identify cell types based on known markers^9,12^. Cells in cluster 25 were removed from the dataset and the subsequent downstream analysis due to the potential of doublets that were forming this cluster. The remaining 34 distinct cell clusters were grouped into seven major cell types, including myeloid immune cells, lymphoid immune cells, fibroblast, Schwann cells, malignant cells, endothelial cells, and pericytes.

#### Subtype integration and clustering

Based on the assignment of major cell type from the manual annotation, cells that belonged to 1) immune cell type (myeloid and lymphoid immune cells together), 2) endothelial and pericytes, and 3) fibroblast, Schwann and malignant cells were respectively taken into re-integration and subclustering in Seurat using the wrapper Harmony^66^. Marker genes of each cell cluster were identified with the Seurat function FindAllMarkers in the “RNA” assay. The top highly expressed and the most differentially expressed marker genes were used to manually annotate the cell types based on prior knowledge and their overlaps with the canonical markers.

#### Marker identification and module score calculation

For each cell type identified, the top 50 most highly expressed (ranked first by avg_log2fold change and then by p_val_adj) marker genes were retained as the markers (**Supplementary Table 2**). The Seurat function “AddModuleScore” was used to calculate the average expression of the marker genes of each cluster. The “Module Activity” of each cluster in each cell was added as a new metadata in the corresponding object and used to visualize in t-distributed stochastic neighbor embedding (t-SNE) plots. Finally, “Module Activity” of cells in any given cluster was compared among the three pathological stages and this comparison was visualized in violin plots.

#### Cell proportion calculation and visualization

In each object (the reference dataset and each re-integrated major cell types), the cell frequencies of each annotated cell cluster were calculated within each pathology. The depletion and enrichment of each cell cluster compared to PN (enrichment score) were calculated using the Pearson residual as below:

$$\text{Pearson}\;\text{residual}=\frac{{\text{Frequency}}_{\text{obs}}-\;{\text{Frequency}}_{\text{exp}}}{\sqrt{{\text{Frequency}}_{\text{exp}}}}$$

Here the Frequency_obs_ is the cell frequency of each cluster in AN or MPNST. The Frequency_exp_ is the cell frequency of each cluster in PN. The enrichment score of each cluster in PN were set to 0. The enrichment scores compared PN (AN vs PN and MPNST vs PN) of particular cell clusters were visualized in a “dot plot”, in which the color indicates enrichment (red) / depletion (blue) and the size of the dot indicates the calculated score. Note that the color scale was set between − 50% and 50% so any scores fell out of this range were manually set to the lower or upper limit for visualization purposes.

#### Inference of copy number variation

The gene expression matrix was used in the software inferCNV (https://github.com/broadinstitute/inferCNV) to predict copy number variations (CNV) in cells. Cells in the clusters that belong to the immune cell compartment and endothelial cell and pericyte compartment were used as reference. Cells that belonged to the fibroblast, Schwann cell, and malignant cell clusters were used as query in inferCNV using the following parameters: cutoff = 0.1, cluster_by_groups = F, cluster_references = F, denoise = T, analysis_mode=‘subclusters’, hclust_method=‘ward.D2’, tumor_subcluster_partition_method=‘leiden’, tumor_subcluster_pval = 0.05, HMM = T.

### Label transfer and cell type prediction

An additional 15 NF1-associated peripheral nerve sheath tumors were processed, captured, and sequenced on a 10x platform as described in the “Single-cell RNA sequencing” section. For each newly sequenced samples, a query object was generated in Seurat using the standard pipeline without integration. Data from additional 11 benign NF1-associated peripheral nerve sheath tumors and 9 MPNST (GSE179033 and GSE207399) was also processed in Seurat using the standard rPCA integration pipeline and used as a query dataset. Each query dataset (from 19 newly sequenced individual tumors and 20 individual publicly available datasets of 20 tumors) was compared to the NF1 tumor reference data, which was an integrated dataset of 55 tumors of all pathological stages. Anchor based label transfer using the NF1 tumor reference was performed to predict the cell type for each cell in each of the query dataset using the Seurat functions FindTransferAnchors and TransferData.

### Inferred copy number variation

For each above-mentioned query dataset, copy number variation was predicted using inferCNV. Based on the result of label transfer, the predicted fibroblast, Schwann cells, and malignant cells were considered as query to be compared with the reference cells (predicted pericyte, immune and endothelial cells) in inferCNV using the following parameters: cutoff = 0.1, cluster_by_groups = F, cluster_references = F, denoise = T, analysis_mode=‘subclusters’, hclust_method=‘ward.D2’, tumor_subcluster_partition_method=‘leiden’, tumor_subcluster_pval = 0.05, HMM = T.

### Malignant cell identification

Upon label transfer, a cell was identified as a potential malignant cell if it was annotated as one of the “Malignant cell 1”, “Malignant cell 2”, or “Bridging malignant cell” type based on transcriptional similarity. The total number of potential malignant cells of each sample was calculated as the summary of cell numbers of the three malignant cell types.

### Malignant score calculation

For each predicted malignant cell, a malignant score was calculated with the consideration of 1) the confidence of the prediction based on its transcriptional similarity from the label transfer, and 2) the level of aneuploidy calculated based on the inferred copy number variation from the results of inferCNV. Each cell was given an initial malignant score of 1 if it was identified as malignant as described above or a score of 0 if it was identified as any other cell types. We next corrected this malignant score by multiplying it with “prediction.score.max” from the result of label transfer. We further derived cell-level cnv score from the file “17_HMM_predHMMi6.leiden.hmm_mode-subclusters.infercnv_obj” from the inferCNV result. For each cell, the cnv score was calculated using the following formula:

$$\text{cnv}\;\text{score}=\sqrt{\sum {\left(\frac{\;\text{infercnv\_object}@\text{expr}.\text{data}-3}{2}\right)}^{2}}$$

In each individual samples, the malignant score of each predicted malignant cell was calculated as:

malignantscore=1×prediction.score.max×cnv.score

### Immunohistochemistry staining and analysis

Formalin-fixed, paraffin-embedded tissue blocks from tumor specimens were cut into serial 5μm thick sections and mounted on plus-charge glass slides. The serial sections from each specimen were stained for hematoxylin and eosin (H&E) and antibodies to CD8 and FOXP3. Automated dual staining was performed on Leica Bone RX (Leica Biosystems) and detection was performed using the Bond Polymer Refine Kit (DS9800, Leica Biosystems). Each slide was counterstained, baked, and cover-slipped using EcoMount. The single stained slides were scanned using the Hamamatsu Nanozoomer to a magnification of 20x (ScanScope XT) and analyzed using HALO imaging analysis software (Indica Labs). The HALO digital analysis software was used to calculate intratumoral immune cell counts and tissue area. For each slide, we used the same algorithm to calculate the immune cell density and manually reviewed each case to ensure consistency and accuracy.

On the HALO software platform, each stained slide was imported as .ndpi files and deconvoluted using the Deconvolution Analysis algorithm to obtain a single .decon files. Each .decon file contained two channels including a channel with specific marker staining and another channel with the nuclear staining (hematoxylin). For each specimen, the related .decon files were registered and then fused into a single .tiff file that contained all the channels from each individual .decon file, aligned with each other. Each channel is assigned a pseudocolor. This analysis allowed for the alignment and overlay of the serial sections of the same tumor specimen into a single merged file, allowing for analysis of co-localization of the immunohistochemistry stained markers.

### Protein proximity extension assay

Proximity extension assay in combination with next-generation sequencing for high-throughput proteome-wide analysis (Olink assay) were performed as described in detail previously^67^. Briefly, 40uL of plasma from 118 samples (healthy (n = 10), PN (n = 29), atypical neurofibroma (AN; n = 25) and MPNST (n = 54)) underwent immunoassay for relative abundance of 1,463 proteins using dual-recognition antibodies labelled with unique DNA oligonucleotides (four 384-plex Olink panels: inflammation, oncology, cardiometabolic, and neurology). Relative concentrations of assayed proteins were readout by next-generation sequencing with outputs in normalized protein expression (NPX), logarithmically related to protein concentration. Protein values below the limit of detection (LOD) were replaced with the LOD divided by the square root of 2 and each protein was rescaled to have a mean of 0 and a standard deviation (SD) of 1^68^. Differentially expressed proteins were identified using one-versus-one disease state comparisons with ANOVA and post-hoc Tukey HSD of NPX values. Proteins significantly enriched in MPNST plasma (*p* < 0.05, delta NPX > 0) relative to PN were filtered for only proteins encoded by genes overexpressed in malignant cell and regulatory T-cell (Treg) clusters in the NF1 scRNAseq. Filtered proteins’ individual performance at identifying MPNST were assessed using Youden’s Index and receiver operating characteristic (ROC) curve in one-versus-all (OVA) comparisons.

### Olink pan-cancer protein markers

Two hundred and twenty six non-leukemia or lymphoma cancer-associated circulating proteins were curated from Papier et al.’s published pan-cancer Olink dataset, which examined 19 cancers (head and neck, esophagus, stomach, colorectum, liver, pancreas, lung, malignant melanoma, breast, uterine, ovary, prostate, kidney, bladder, brain, thyroid) in 54,306 participants from the UK Biobank (*40*). Our NF1 specific dataset was filtered to contain only proteins contained in this published list. The performance of these individual proteins at identifying MPNST was assessed in our dataset using Youden’s Index and receiver operating characteristic (ROC) curve in one-versus-all (OVA) comparisons.

### Integrated Protein Signature and Recursive Feature Elimination

Multiple proteins were integrated using a support vector machine (SVM) model (R package e1071 (https://cran.r-project.org/web/packages/e1071/index.html). The best performing SVM kernel (linear, radial, polynomial, sigmoid) was determined using leave-one-out cross-validation with all 50 candidate biomarker proteins. The kernel yielding the highest accuracy while maintaining AUC > 0.8, radial kernel (linear kernel: accuracy = 0.847, AUC = 0.944; radial kernel: accuracy = 0.915, AUC = 0.967; polynomial kernel: accuracy = 0.898, AUC = 0.930; sigmoid kernel: accuracy = 0.898, AUC = 0.966), was selected for all subsequent SVM models and analyses. The cost parameter (C) in the SVM model was set to 1 to avoid potential overfitting given our limited sample size. Median probabilities of a 10 repeat, 5-fold cross-validation SVM were termed the MPNST probability score. Performance of the integrated protein model was assessed using Youden’s Index and a ROC curve of MPNST probability OVA scores.

## Supplementary Material

Supplementary Files

This is a list of supplementary files associated with this preprint. Click to download.

- SupplementaryTable1.Cohortofpatientspecimens.xlsx
- SupplementaryTable2.Top50genemarkersinforeachsinglecellcluster.xlsx
- SupplementaryTable3.Cohortofplasmasamplesprofiledbyproteomics.xlsx
- SupplementaryTable4.GOtermsfromenrichmentanalysisof127scRNAseqidentifiedcandidateMPNSTmarkers.xlsx
- SUPPLEMENTARYFIGURES.docx

## Figures and Tables

**Figure 5:**
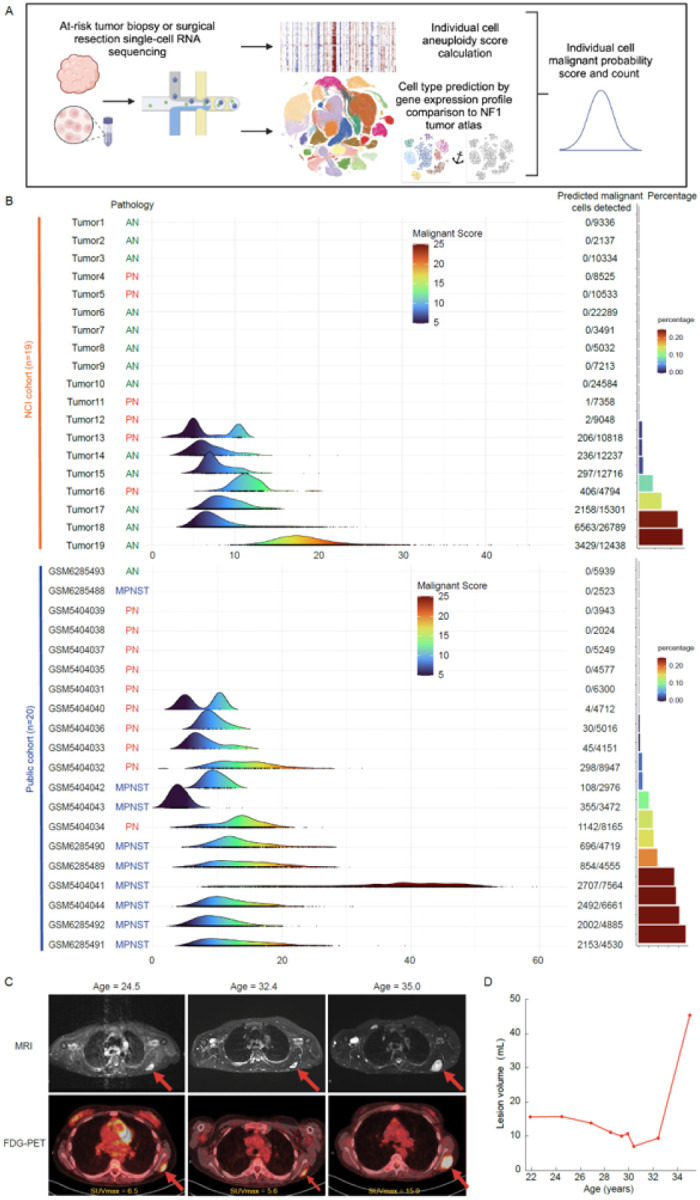
Prediction of malignant transformation from scRNAseq. **A.** Diagram showing the process of using a single-cell NF1 nerve sheath tumor reference to predict malignant transformation. **B.** Ridge plot shows the malignant score of predicted malignant cells in an independent cohort of 19 NF1 nerve sheath tumors from patients seen at the Clinical Center of the NCI (top) and publicly available single-nuclei RNAseq data of 20 NF1 nerve sheath tumors (bottom). **C.** Magnetic resonance imaging of a tumor monitored longitudinally. **D.** Volumetric measurement of the tumor shows exponential growth prior to the diagnostic biopsy.

## Data Availability

All generated scRNAseq data was deposited to dbGaP with the accession number phs003519. Proteomic data are available through Synapse SynID syn51115822 (https://doi.org/10.7303/syn51115822).
